# Managing Decompression Sickness in a Low‐Resource Setting, a Clarion Call to Improve Systems: A Case Report

**DOI:** 10.1155/carm/4030617

**Published:** 2026-06-09

**Authors:** Fonseka C. L., Liyanage D. C., Nusaiha M. N. A., Nawagamuwa L. P. R. S., Nanayakkara B. P. N., Ruben J., Ariyadewa D. K., Dissanayake A. S.

**Affiliations:** ^1^ Department of Medicine, Faculty of Medicine, University of Ruhuna, Karapitiya, Galle, Sri Lanka, ruh.ac.lk; ^2^ University Medical Unit, National Hospital Galle, Karapitiya, Galle, Sri Lanka; ^3^ Diving and Hyperbaric Medicine Unit, Navy General Hospital-Welisara, Sri Lanka Navy, Colombo, 11010, Sri Lanka; ^4^ Department of Parasitology, Faculty of Medicine, University of Ruhuna, Karapitiya, Galle, Sri Lanka, ruh.ac.lk; ^5^ Diving and Hyperbaric Medicine Unit, Director General Health Services Office, Colombo, Sri Lanka; ^6^ Sri Lanka Navy Headquarters, Defence Headquarters Complex, Baththaramulla, Colombo, Sri Lanka

**Keywords:** case reports, decompression sickness, hyperbaric oxygenation, neurologic manifestations, Sri Lanka, travel

## Abstract

Decompression sickness (DCS) is a life‐threatening clinical condition that occurs following an uncontrolled rapid ascent after a deep dive, leading to the formation of gas bubbles in the blood occurring due to the rapid decline in environmental pressure. We report the case of a 32‐year‐old male who presented with bilateral lower limb numbness eight hours following a diving session. He was diagnosed with neurological DCS and transported 365 km to the Naval Hospital, Eastern Command where he received hyperbaric oxygen treatment and made a full recovery. This individual developed DCS after repetitive dives within a week despite having planned ascents with advice from a diving instructor. He switched between different rented diving computers; consequently, these devices lacked cumulative dive history to indicate minimum surface time required for sufficient nitrogen gas washout. Both the diver and instructors had a suboptimal assessment of the cumulative dive exposure and worked with a false reassurance of having planned ascents under supervision. In addition, this report highlights critical delays in the diagnosis of DCS and complexities involved in accessing the country’s only hyperbaric treatment facility. It also emphasizes the need for empowering diving centers and medical professionals for early identification and facilitating access to prompt treatment.

## 1. Introduction

Decompression sickness (DCS) is a potentially life‐threatening clinical condition that occurs following a rapid ascent after a deep dive, leading to the formation of nitrogen gas bubbles in the blood due to the precipitous decline of environmental pressure from a high‐pressure environment. Type II DCS is the severe form, characterized by cardiorespiratory and neurological manifestations such as numbness, weakness, paralysis, or coma. Although modern safety protocols have reduced the overall incidence of DCS in sport diving to approximately 3 cases per 10,000 dives, the risk remains strongly correlated with cumulative dive exposure and is 2.5 times higher in males [1]. Recent large‐scale epidemiological analyses estimate an overall DCS incidence rate of approximately 0.49%, identifying inadequate surface intervals as a critical independent predictor of injury [2]. The only definitive treatment for DCS is hyperbaric oxygen treatment (recompression therapy), where the patient is placed in a pressurized chamber. During hyperbaric oxygen treatment, the gradual decompression of ambient pressure reduces bubble volume and facilitates the resorption of nitrogen into the bloodstream, where it is transported to the lungs and eliminated via exhalation [3]. This is vital in Type II DCS, where timely interventions are necessary to prevent permanent neurological sequelae. The previous two case reports from Sri Lanka (from 1986 to1995) describe divers who had permanent neurological disability due to a hurried ascent in response to technical problems and had no decompression treatment due to lack of availability [4, 5].

Globally, DCS remains a significant health concern among the diving population, gaining increasing attention due to expanding recreational diving tourism. Similarly in Sri Lanka, the growing popularity of recreational diving has resulted in an increase in the number of DCS cases. This case highlights a case of Type II DCS which occurred after repetitive dives without adequate surface intervals even when ascents were well‐planned and accompanied by a diving instructor. This also emphasizes the importance of assessing cumulative diving history and focuses on the challenges in accessing hyperbaric oxygen therapy (HBOT) in Sri Lanka.

## 2. Case Presentation

A 32‐year‐old, previously healthy male from Sweden developed bilateral lower limb numbness 30 min after surfacing from a recreational dive at Unawatuna, Galle, Sri Lanka, and subsequently presented to the hospital eight hours later. He had completed 18 self‐reported diving sessions over the previous week, including two dives the day before the incident. The patient did not have adequate surface intervals between dives, preventing sufficient nitrogen washout. Such high‐frequency diving is not uncommon in Unawatuna, which hosts over 30 commercial dive sites, prompting tourists to routinely request two to three dives per day to maximize their experience. Further, in these seasonally overcrowded settings, assessing precise surface interval durations may be overlooked, as divers are ambitious to explore a maximum number of diving sites and the rapid turnarounds of divers in diving centers may hinder assessment of the cumulative diving history. On the day of the incident, he completed a 26 m dive lasting 25 min. He dove with a diving instructor using a wrist‐mounted diving computer. However, he used different devices at various centers during the week. His ascent to surface was as instructed by the diving instructor and guided by the diving computer. After 30 min of surfacing, he developed numbness and weakness of his lower limbs, and possible DCS was suspected. At the diving center, oxygen was administered via a face mask. As there was only slight symptomatic improvement without resolution of major neurological deficits, he was transferred to the hospital nearly 7 hours after symptom onset. In addition, they were seeking assistance from overseas doctors and the Naval Hospital Eastern Command to find the best way to seek hyperbaric treatment, as it is located 365 km away from Galle. This led to a cumulative delay to admit to the hospital. He was admitted to National Hospital Galle (NHG) 8 h following the diving session. Upon admission, he was conscious and rational and had stable vital parameters and normal oxygen saturation. While he denied pain, he reported bilateral lower limb weakness and numbness. On examination, the tone was increased bilaterally, and the knee and ankle reflexes were exaggerated with a mild lower limb weakness. There was a sensory level at T10 with impaired pain and touch sensations. There was no spinal tenderness. Bowel and bladder functions were fully preserved, with no urinary retention or incontinence observed during the hospital stay. Although DCS was the most probable diagnosis, his symptoms were initially attributed to anxiety, as the sensory symptoms and subtle hyperreflexia can be subjective in an anxious individual, and due to the assumption that he had no evidence of spinal trauma. Due to the unfamiliarity of managing DCS, especially having not encountered Type II DCS cases before by the hospital team, there was an initial transient uncertainty of diagnosis. The attending medical professionals expected the patient to have hypoxia rather than a neurological deficit on presentation, mimicking a spinal cord injury. However, without much delay, relevant material available online including management guidelines of DCS from Sri Lanka were referred and clinical management was realigned. Initially, an urgent MRI scan of the spine was planned but was soon abandoned due to a lack of a history of spinal trauma and due to the system delays in obtaining an MRI of the whole spine. Hence, definitive management on strong clinical grounds was sought.

He was treated with 100% oxygen via a nonrebreather face mask and continuous infusion of intravenous fluids. Since the only definitive treatment facility with a hyperbaric chamber was located 365 km away, arrangements for immediate transfer were initiated. Air transfer was the optimal transport modality, and such helicopter facilities are available commercially from the Sri Lanka Air Force. As the helicopter is nonpressurized, low‐altitude flight is required to prevent further decompression. Due to the late hours (23:00 h) and poor visibility, a low‐altitude night flight was deemed unsafe, and waiting till daylight for use of such a facility would have caused further delays. Hence, ground transport via hospital ambulance was arranged. He remained hemodynamically stable enroute and was admitted to the Naval Hospital Eastern Command after 9 hours of travel. He underwent HBOT in accordance with established Undersea and Hyperbaric Medical Society (UHMS) guidelines [6]. Treatment was administered using the US Navy Treatment Table 6 (USN TT6 is currently considered the “standard of care“ for the treatment of decompression illness), selected according to his dive profile and clinical parameters [7]. Two treatment cycles were conducted, and following the second cycle, his symptoms completely resolved, and he was discharged after two days, with a normal neurological examination. Upon discharge he was instructed to avoid flying for the next 72 h, to avoid diving, and to avoid smoking and alcohol.

## 3. Discussion

Sri Lanka has a vibrant tourism industry, establishing itself as the country’s third‐largest source of foreign exchange earnings, generating 3.2 billion USD in 2024. [8]. Popular diving destinations are scattered around the country’s coastal belts, drawing both local and overseas divers. However, Sri Lanka relies on a single operational hyperbaric chamber, located on its northeastern coast. This poses a significant logistical challenge, as the majority of the recreational diving sites such as Hikkaduwa, Mirissa, and Unawatuna are located over 365 km on ground travel from this center. The HBOT center at the Sri Lanka Navy’s diving and salvage unit located at Trincomalee was established as a ten‐person, two‐lock hyperbaric chamber in 1987, and since its establishment, approximately 120 individuals have been treated for DCS.

The risk of DCS has decreased with the use of modern equipment, such as wrist‐mounted diving computers, which provide practical decompression protocols for divers [9]. Dive computers utilize decompression algorithms to mitigate the risk of decompression illness by continuously updating depth profiles and recalculating decompression variables, including maximum depth, diver age, surface intervals, and total breathing gas consumption [10]. They also account for starting altitude, water temperature, and freshwater or seawater conditions, though some still require manual input. The Bühlmann algorithm and newer algorithms like the Reduced Gradient Bubble Model (RGBM) and the Varying Permeability Model (VPM) are used in these computers. These newer models operate on the premise that asymptomatic microscopic bubble nuclei (or ‘seeds’) exist in blood and tissues and will expand upon decompression; thus, adjust decompression schedules to keep the external pressure large and the inspired inert gas partial pressures low during decompression [11]. Despite these technological advancements, no algorithm can guarantee protection against DCS. These devices cannot account for individual risk factors like age, body composition, dehydration, alcohol consumption, or cardiac shunts. This limitation is rooted in the historical development of these tables using young male military personnel in superb physical condition as their primary test subjects. Consequently, when modern dive computers are used for variable physiological profiles of today’s recreational divers, they often show significant margin errors [10, 12].

With each dive, clinically insignificant levels of gas bubbles are formed. The excess nitrogen remains dissolved in the body tissues for at least 12 h after each dive [10]. Hence, repeated dives within 1 day are more likely to cause DCS than a single dive. For repetitive dives, a single continuous diving computer would calculate the minimum required surface interval as well as the depth of the next dive. In this case, the patient’s use of different computers from various diving centers resulted in the loss of critical cumulative dive history, which was a key factor in the development of DCS. Therefore, it is recommended that divers consistently utilize a single device for all repetitive dives. When possible, carrying a secondary backup computer is advisable to ensure that cumulative dive logs and nitrogen wash‐out histories are preserved in the event of equipment failure.

The uniqueness of this case lies in the convergence of diver‐driven behavioral oversights, where it is desired to maximize the diving experience during the holiday season and diver burden in diving centers along with commercial pressures. The practice of renting different dive computers systematically masks the patient’s cumulative nitrogen history; thus, rather than preventing injury, the technology provided a false sense of security. This risk can be amplified in tourist‐dense areas where high turnover of divers gives inadequate time to explore the cumulative diving history, often overriding the safety limits.

This specific intersection of behavioral hazards and logistical barriers is rarely captured in contemporary literature. A review of recently reported literature between 2025 and 2026 of seven case reports and two case series of DCS are summarized in Table 1 [13–21]. Four out of the seven case reports and the case series including 13 cases had neurological symptoms suggestive of Type II DCS, the majority from high‐income countries. Though three of the case reports and one patient in the case series had residual deficits, our patient achieved full recovery despite a delay in treatment. Importantly, these published cases generally reflect well‐resourced environments and overlook the profound logistical challenges of managing DCS in low‐ and middle‐income countries (LMICs), many of which are popular diving destinations that experience heavy seasonal overcrowding. In our setting, we identified several critical challenges to optimal management. Initial presentation was delayed by 8 h at the dive center due to a lack of awareness of when to seek medical care because there was partial improvement of symptoms with oxygen. In addition, misplaced confidence due to planned ascents with dive instructors and rented computers contributed to a denial of the diagnosis. Due to the lack of education and inexperience of managing cases of Type II DCS, there was an insignificant delay in arriving at the diagnosis on admission. Beyond clinical challenges, the patient acknowledged that he experienced prolonged uncertainty in acquiring specific treatment in a low‐resource setting, leading to significant psychological distress when seeking medical care. Once DCS was diagnosed, transferring the patient to the nearest HBOT facility which is located seven to 8 hours away from Galle, presented a major logistical hurdle. Air transfer, which theoretically offers the fastest transit, was not feasible as the available helicopters were unpressurized, and low‐altitude helicopter flights are restricted during night transfers due to poor visibility. Consequently, ground transport remained the only viable option, further complicated by having to avoid hilly terrains as altitude changes could exacerbate the patient’s neurological condition.

**Table TABLE 1:** Summary of published case reports or case series on decompression sickness (DCS) in 2025 and 2026.

Study and year (ref)	Design (n)	Country	Patient profile	Comorbidities	Exposure profile	Clinical presentation	Imaging	Time to treatment	Intervention	Clinical outcome
Mason et al., 2025 [13]	Case Report (1)	Australia	28/F (44 previous dives)	Oral contraceptive user	12 m of seawater (msw) or 89 min 24 h later 35 msw, 44 min and second dive 26 msw for 30 min omitted decompression	Neurological DCS	Brain MRI: Multifocal embolic infarcts. Transthoracic echocardiogram (TTE)/trans esophageal echocardiogram (TOE): 6 mm patent foramen ovale (PFO) and severe right ventricular dilatation.	3.6 h	USN (US Navy) Table 6 (initial) then USN TT5; 22 total Hyperbaric Oxygen Therapy (HBOT) sessions	At 6 months residual deficits (persistent paresthesia of legs with balance issues)
Fernandez‐Cisneros Alejandro, 2026 [14]	Case Report (1)	Philippines	55/M	Obese, hypertensive	Max 22.6 m (3 dives) short surface intervals	Neurological, Cutaneous, Cardiopulmonary DCS	Abdominal CT: Portal venous gas in liver, brain computed tomography (CT scan) and chest X‐ray showed no abnormalities. Follow‐up TTE: Positive for Patent Foramen Ovale (PFO).	Required transfer. Time not recorded.	USN Table 6; IV fluids, norepinephrine	At 12 months follow‐up residual deficits (hearing, improved gait)
Wang et al., 2025 [15]	Case Report (1)	China	51/M (Com)	Hypertension, Type 2 diabetes mellitus, obese, alcohol consumption (> 100 g white liquor) night before dive	19, 120 min. Heavy lifting under water. After completing the work, the diver underwent in‐water staged decompression, which included two stops: the first at 6 m for 8 min, and the second at 3 m for 26 min. The entire decompression process lasted 45 min	Cardiopulmonary and Musculoskeletal DCS	CT: multiple gas emboli in the portal vein branches, superior mesenteric vein, inferior vena cava, pulmonary artery, and right ventricle, along with several intra‐abdominal gas shadows. TTE: Negative for PFO. repeat abdominal CT confirmed complete resolution of the gas emboli	2.0 h	Chinese Naval Medical University Table IV	Full recovery
Kamiimabeppu et al., 2025 [16]	Case Report (1)	Japan	63/M (Instructor)	Hypertension and previous DCS (6 years prior)	55 m	Musculoskeletal DCS	CT: Intravenous gas in portal, superior mesenteric, and femoral veins. Day 2 CT: Pneumatosis intestinalis and poor enhancement of sigmoid colon/rectum.	Exact not mentioned	HBOT; emergency bowel resection (colostomy)	Complications resolved; transferred
Schmitz, 2025 [17]	Case Report (1)	Costa Rica	35/M (Rec with 71 lifetime dives)	chronic cannabinoid use (3–5 times/week)	14–20 m Multiple dives over 7 days	Neurological, vestibular & cardiopulmonary DCS	ECG: Sinus bradycardia, 2nd‐degree sinoatrial node exit block. Echocardiography: Normal EF (62%).	16.0 h	USN Table 6	Full recovery
Lin et al., 2025 [18]	Case Series (13)		Freedivers					Up to 72 h	In‐water recompression (IWR) (5–25 m); followed by HBOT in some	Most resolved; single permanent deficit
Sugimura et al., 2026 [19]	Case Series (3)	Japan	28/M, 55/M, 52/F		Depths ranging 30–50 m				USN Table 5 (*n* = 1); USN Table 6 (*n* = 2)	Full recovery
Belgen et al., 2025 [20]	Case Report (1)	Cyprus	65/M		35 min exposure	Neurological DCS		Immediate	HBOT; intensive physiotherapy	Residual deficits (clinical improvement)
Wilmshurst et al., 2025 [21]	Case Report (1)	United Kingdom	43/M (Com for 16 years)		17 m of seawater, 160 min	Cutaneous DCS	Bubble contrast TTE: Large atrial right‐to‐left shunt (PFO).		PFO closure	
Our Case	Case Report (1)	Sri Lanka	32/M (Rec)	None		Neurological DCS			USN Table 6	Fully recovered

In contrast, some of the other nations in the region that heavily rely on marine tourism such as Indonesia, Malaysia, and Philippines operate at least five diver‐focused hyperbaric chambers attached to naval or tertiary hospitals, ensuring timely access to treatment. Sri Lanka’s closest neighbor Maldives, offers its divers several privately managed hyperbaric chambers specifically concentrated around popular diving sites. Despite substantial costs involved, establishment of a similar hyperbaric treatment facility, preferably in the Southern coast in Sri Lanka, where many of the diving centers are located is likely to both prevent death and long term neurological sequelae for those with DCS as well as contribute to strengthening diving‐related tourism efforts of the country.

Mitigating these risks requires targeted education across both the diving industry and local healthcare systems. Dive operators must prioritize adequate surface intervals over rapid commercial turnarounds and actively instruct tourists to use a single, continuous dive computer or preferably two to preserve cumulative logs. Concurrently, healthcare workers in coastal areas require focused training to maintain a high index of suspicion for DCS, even when exact dive histories are missing.

## Funding

The authors received no financial support for the research, authorship, and/or publication of this article.

## Consent

Written informed consent was obtained from the patient for publication of this case report.

## Conflicts of Interest

The authors declare no conflicts of interest.

## Data Availability

All available data are mentioned in the case report. Data sharing is not applicable to this article as no datasets were generated or analyzed.
